# 3D-printed shoe soles with structural properties towards reducing knee adduction moments during walking

**DOI:** 10.3389/fbioe.2026.1734486

**Published:** 2026-01-20

**Authors:** Ziang Jiang, Paciane Bo Studer, Matthias Zäh, Christian Kryenbühl, Tingyu Wang, William R. Taylor, Qiang Zhang

**Affiliations:** 1 Institute for Biomechanics, ETH Zürich, Zürich, Switzerland; 2 Swissbiomechanics AG, Einsiedeln, Switzerland

**Keywords:** 3D printing, gait analysis, joint kinetics, knee osteoarthritis, orthosis, shoe soles

## Abstract

**Purpose:**

Lateral wedged insole (LWI) is a representative foot orthosis for the conservative management of knee osteoarthritis. However, recent research indicates the heterogeneity of LWIs’ biomechanical effectiveness, indicating limitations in conventional LWI design and the need to develop new orthoses with improved effects. This study evaluated the efficacy of custom-designed 3D-printed shoe soles in reducing knee adduction moment (KAM), compared with conventional 5° LWIs.

**Methods:**

Shoe soles were 3D-printed with a gyroid infill structure to allow adjustable stiffness, generating a variable stiffness (VS) sole with lateral-medial stiffness differentiation, and a soft heel (SH) sole incorporating the same differentiation plus a soft heel. Twenty-one healthy adults performed gait assessments wearing control shoes with neutral insoles, control shoes with 5° LWIs, VS, or SH shoes. Kinematic data and ground reaction forces (GRFs) were captured. Gait parameters, joint kinematics and kinetics were analysed. Repeated measures ANOVA assessed footwear effects, and multiple linear regression identified key contributors to KAM variation. Statistical significance was set at P < 0.05.

**Results:**

KAM varied significantly across footwear. SH shoes reduced 1st and 2nd peak KAMs by 10.5% (*P* = 0.001) and 8.6% (*P* = 0.032); VS shoes reduced the 2nd peak by 9.1% (*P* = 0.014). Both VS and SH reduced knee adduction angular impulse (KAAI) by 9.7% and 11.8% (*P* < 0.001). 5° LWIs only reduced 1st peak KAM by 7.9% (*P* = 0.009). GRF–knee lever arm was the main contributor to 1st peak KAM changes in VS shoes, while SH shoes’ effects involved both GRF and lever arm. For 2nd peak KAM, the lever arm was dominant contributor; for KAAI, lever arm and stance time were main contributors.

**Conclusion:**

3D-printed soles reduced KAM variables more effectively than 5° LWIs, supporting their potential for biomechanically optimised footwear upon further assessments on individuals with knee osteoarthritis.

## Introduction

Knee osteoarthritis (KOA) is one of the primary causes of knee pain and functional disability in elderly individuals (Van Dijk et al., 2008), reported to affect 22.9% of the global population over the age of 40 years in 2020 (Cui et al., 2020). As there is no current cure, over 50% of patients with end-stage KOA undergo costly knee arthroplasty (Weinstein et al., 2013), imposing a substantial socioeconomic burden on global healthcare systems. Conservative treatments are deemed valid approaches for early management of KOA to alleviate symptoms and retard irreversible joint damage (Im, 2022; Kanamoto et al., 2020).

Foot orthoses are one of the plausible non-pharmacological options endorsed by the Osteoarthritis Research Society International (McAlindon et al., 2014; Bannuru et al., 2019). Various foot orthoses have been presented (Erhart-Hledik et al., 2021; Madden et al., 2017; Haim et al., 2012), primarily aiming to modify and reduce the impact of loading on arthritic knee cartilage. Their rationale stems from the reported association between increased knee loading (knee moments and angular impulse) and higher risk of KOA progression and pain (D'Souza et al., 2022; Hutchison et al., 2023). For example, for varus limb alignment, the distribution of joint contact loads towards the medial tibiofemoral compartment during daily functional activities are believed to overload the medial condylar cartilage and accelerate the progression of KOA in this region (D'Souza et al., 2022; Favre and Jolles, 2016). Considering the predominant proportion of varus-associated medial KOA cases (Dearborn et al., 1996), inexpensive lateral wedged insoles (LWIs) (Hinman et al., 2012; Esfandiari et al., 2020) have become one of the most widely utilised foot orthoses for the conservative management of clinical KOA. By altering the frontal-plane angle of the ankle/subtalar joint and laterally shifting the foot centre of pressure (COP) (Almeheyawi et al., 2021), LWIs have been shown to successfully reduce external knee adduction moments (KAMs, a widely used surrogate of medial knee loading) by approximately 4%–10% in individuals with medial KOA during walking (Hinman et al., 2012; Kakihana et al., 2005). However, their clinical efficacy on KOA remains inconclusive (Parkes et al., 2013), evidenced by unclear symptomatic or structural improvements after long-term usage (Bennell et al., 2011). One reason might be the heterogeneity of LWIs’ biomechanical effectiveness (McAlindon et al., 2014; Lewinson and Stefanyshyn, 2016), given that 13%–23% of patients experience an adverse increase in KAM while walking with LWIs (Lewinson and Stefanyshyn, 2016; Hinman et al., 2008). These findings highlight limitations in LWI design that may compromise their therapeutic efficacy for KOA.

Other developments to manage KOA include orthotic footwear such as variable stiffness shoes with a stiffer lateral midsole (Erhart-Hledik et al., 2021) or a rocker-soled shoe design (Madden et al., 2017), which have been shown to lower the KAM (Madden et al., 2015) and have even been associated with reduced knee pain (Erhart-Hledik et al., 2012). In general, the KAM exhibits a double-bump pattern, with a peak occurring at both the early and late stance phases of gait (Khalaj et al., 2014), but it is often unclear where and when these therapies are most effective. This lack of clarity plausibly explains the observed variations in KAM outcomes between individuals, even with the same intervention. One concept to modify both the 1st and 2nd KAM peaks has been presented in the APOS-System shoe, which features two biomechanical elements attached beneath the sole that can be adjusted to modify the COP (Bartels and Suk, 2022). This adjustable system has been reported to reduce the 1st peak KAM by 8.4% and the 2nd by 12.7% (Haim et al., 2012). However, its high cost and limited availability in only a few countries hinder its widespread adoption among KOA patients worldwide. Importantly, the presentation of this novel concept has opened perspectives on approaches to improve biomechanical efficacy throughout the different phases of gait, and also account for the individual characteristics of KOA patients (Kim et al., 2018). This concept also highlights the different contributions of the 1^st^ and 2^nd^ peak KAMs (Baniasad et al., 2022), providing a basis for implementing multiple orthotic concepts towards improving the overall therapeutic efficacy of orthoses, as well as modifying different characteristics of the KAM (1st peak, 2nd peak, and knee adduction angular impulse (KAAI)). However, the need to develop novel orthotic shoes that integrate distinct functional components at different regions of the sole remains unmet. Here, advanced manufacturing approaches such as 3D printing offer great potential in producing shoe soles embedded with continuously varying stiffness and geometry, especially given the high structural and morphological freedom.

Presented here is a 3D-printing approach for manufacturing novel shoe soles with orthotic functionality. Using this approach, two types of shoes were developed, each with different functional components at the hind-foot and fore-foot sole regions. In addition, the current study examined the biomechanical efficacy of these shoes for reducing KAM variables during walking, compared to control shoes and 5° LWIs. The changes of other biomechanical measures such as the ground reaction force (GRF) and its lever arm were also studied in the context of identifying the mechanism of KAM variations with different shoes. It was hypothesized that the 3D-printed shoe soles would exhibit significant reductions in peak KAMs and KAAI during walking over control shoes, with a superior efficacy compared to 5° LWIs.

## Materials and methods

### Design and manufacture of footwear

**FIGURE 1:**
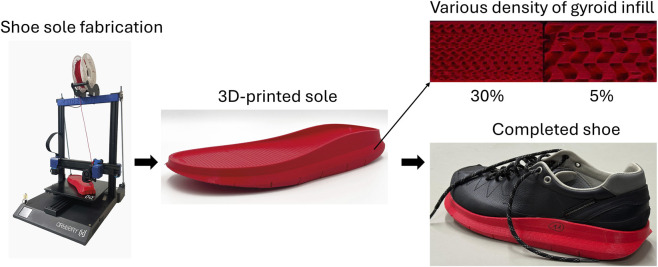
3D-printing fabrication of the shoe soles.

The shoes used in this study to investigate the biomechanical efficacy for modifying KAM consisted of a uniform leather upper and a 3D-printed sole (Figure 1). The soles were designed in Rhinoceros 3D (version: 8, Robert McNeel and Associates, Seattle, United States), containing a neutral midsole base and a functional outsole (Figure 1). The inner structure of the outsole followed a gyroid shape, also known as a triply periodic minimal surface (Silva et al., 2021). This infill structure possesses remarkable density-to-strength efficiency and is easily tuneable, allowing the sole stiffness to be adjusted while maintaining its overall low weight. The sole model was sliced in UltiMaker Cura (version: 5.4.0 Ultimaker B.V., Utrecht, Netherlands) to convert 3D structures into printable layers. A fused filament fabrication 3D-printer (Artillery 3D, Hong Kong) was employed to fabricate the shoe soles, using thermoplastic polyurethane material.

In general, the shoe outsole was discretised into four regions: hind-, mid-, fore-, and toe-regions. Whereas the mid- and toe-regions were printed using a soft design (5% of gyroid infill’s density), the hind- and fore-regions were fabricated using two designs: 1) a variable stiffness (VS) sole with lateral-to-medial ratio of 6:1 (gyroid infill density of 30% and 5%, respectively), with a stiffness separation along the mediolateral mid-line of the outsole; 2) a soft heel (SH) sole with a constant gyroid infill density of 5% in the hind-region, while keeping the fore-regions the same as the VS sole (30% and 5%). Here, the sole’s gyroid infill density was determined based on a trade-off consideration of sole’s structucal robustness and functionality. First, a gyroid infill density of 30% was designed for the stiffer lateral region of the sole. Then, a gyroid infill density of 5% was selected for the softer medial region of the sole. Although a sole with lower gyroid infill density could be designed, any value below 5% would make the sole prone to wear and structural failure under repeated load. The shoe soles were approximately 15–30 mm in thickness, representative of the geometrical shape of a sneaker-type shoe (Koegel et al., 2024). A Shore hardness tester (HBA 100–0, Kern and Sohn GmbH, Balingen, Germany) was employed to characterize the stiffness of the shoe sole. Based on the results of five attempts at each region, an average of Shore A 68.3 and 36.4 were determined at the soles with gyroid infill densities of 30% and 5%, respectively.

Once the shoe soles were fabricated, they were bonded to the shoe uppers, with a neutral insole inserted. In addition to the VS and SH shoes, insoles with 5° lateral wedge were fabricated with ethylene vinyl acetate. A similar sneaker-type shoe with neutral insoles was used as the reference control. Different sized shoes and insoles were prepared to cover the range of the subjects’ foot sizes.

### Subject recruitment

The study sample size was estimated using the G*Power software suite (Version 3.1.3; Heinrich-Heine-Universität Düsseldorf, Düsseldorf, Germany). A previous study on foot orthoses with arch support and lateral wedge reported an effect size (ES) of 0.26–0.58 for comparison of KAM variables in different orthotic designs (Dessery et al., 2017). Submitting the lowest ES (0.26) to a power analysis for repeated measures of analysis of variance (RM-ANOVA) revealed that a minimum of 17 subjects would be necessary to generate an 80% statistical power (α level of 0.05) for detecting biomechanical differences in the current study. Thus, a total of 21 healthy adults (12 females and 9 males; Age: 26.7 ± 6.6 y.o.; BMI: 23.2 ± 2.6 kg/m^2^) were recruited to participate in the study. Individuals were excluded if they: 1) experienced foot or ankle injury in the past 6 months; or 2) had any history of lower-limb surgery. This study was conducted in accordance with the Declaration of Helsinki. Ethical approval was obtained from the institutional Ethics Committee, and each subject provided written informed consent before participation in this study.

### Gait measurements and data collection

Avoiding any exhaustive exercise 24 h prior to the data collection session, all subjects performed level walking tests while wearing different shoes or insoles. A total of 42 reflective markers (Ø = 14 mm) were attached to the body, distributed bilaterally on the subjects’ shoulders, iliac crest, anterior superior iliac spine, posterior superior iliac spine, greater trochanter, lateral and medial femoral epicondyles, lateral and medial tibial malleoli, posterior calcaneus, head of the 1st metatarsal, head of the 5th metatarsal, and base of the 2nd toe, as well as onto the sternum, sacrum, thigh, and shank. A 20-camera optical motion capture system (Vicon Motion Analysis Inc., Oxford, United Kingdom; 200 Hz) and three 3D force plates (Kistler Holding AG, Winterthur, CH; instrumented on the walkway; 2000 Hz) were used to collect the maker trajectory and GRF data.

The subjects performed gait trials under four different footwear conditions: 1) VS shoes; 2) SH shoes; 3) control shoes with 5° LWIs; 4) control shoes with neutral insoles. Before data collection, subjects were allowed familiarization time of approximately 2–3 min walking with each shoe, in order to acclimatise to differences in walking sensation. Before formal measurements, each subject first performed a static calibration trial. The subjects reported their dominant leg, which was defined as their index leg. For each shod condition, the subject performed gait trials until three (McGinley et al., 2009) valid gait cycles (clean index foot strike hits on the force plates) could be recorded for analysis. The walking tests were performed at each subject’s self-selected speed. The four footwear conditions were tested in a computer-generated random sequence, with a washout (rest) period of 5 min between measurement conditions.

### Data processing

Visual3D (HAS-Motion, Ontario, Canada) was used to process the collected data to compute biomechanical outcomes. Raw motion and force data were low-pass filtered (Butterworth, 4th order, bidirectional) with a cut-off frequency of 20 and 200 Hz, respectively. The static trial was used to determine the locations of joint centres and axes of rotation in each subject. Here, the ankle joint centre was identified as the midpoint between the medial and lateral malleoli markers. The knee joint centre was determined as the midpoint of the lateral and medial femoral epicondyle markers, while the hip joint centre was defined using an approach reported by Bell et al. (1990). The external KAM was calculated as the cross product of the frontal-plane GRF and its lever arm to the knee joint centre, and reported in the tibial reference frame (long axis between the ankle and knee joint centres, directed orthogonally to the femoral epicondyle markers) (Madden et al., 2017; Shull et al., 2013). Upon calculating the KAM patterns, its 1^st^ and 2^nd^ peaks were identified as the maximum values in the first and second halves of the gait stance, respectively. KAAI was estimated as the integral of the KAM over the gait stance phase (Thorp et al., 2006). Other functional variables of interest included gait spatiotemporal parameters, lower-limb joint kinematics, and GRF parameters (see Supplementary, Supplementary Table S1). For reporting, joint angles were defined as the rotation of a segment with respect to its nearest proximal segment, and were interpreted using a Cardan sequence, in which the X-axis was directed medial→lateral, the Y-axis posterior→anterior, and the Z-axis distal→proximal. A right-hand rule was utilized to determine rotation polarity, such that knee extension, adduction, and internal rotation were defined as positive. A gait cycle was defined as the period from one heel-strike to the next heel-strike of the same foot, identified using the force plate signal (threshold 10 N). The stance phase of gait was defined as the period from heel-strike to toe-off accordingly. For each footwear condition, the final results were averaged from the data of the three gait cycles measured, and were time normalized where necessary. Knee moment, lever arm, and GRF data were normalized to height (m)*mass (kg), height (m), and weight (N), respectively.

### Statistics

Statistical analysis was conducted using the SPSS software suite (version 28.0; SPSS Inc., Chicago, Uniited States). The significance level (α) was set *a priori* at 0.05. The results were presented as mean ± standard deviation (SD). For the KAM patterns during the stance phase, one-dimensional statistical parametric mapping (SPM1D) was applied node-wise to time-normalised KAM waveforms. Here, one-way repeated measures analysis of variance (SPM1D{F}) was conducted to examine differences in the KAM curves between the control shoe and the other footwear. In the event of a significant main effect, *post hoc* comparisons were conducted between each pair of footwear conditions.

One-way repeated measures analysis of variance (ANOVA) was conducted to examine differences in the variables of interest across all footwear conditions. When a significant main effect was observed, *post hoc* tests with *Bonferroni* correction were conducted to identify pairwise differences. Effect size (ES) was determined using partial eta squared (η^2^) (Richardson, 2011) and classified as: 0.01—small, 0.06—medium, and 0.14—large. To identify the mechanisms of KAM variations resulting from various footwear conditions, multiple linear regression (MLR) analyses were conducted, with Z-scores used to standardise the coefficients. In the first two models, variations in the 1st and 2nd peak KAMs between the VS, SH, and control shoes were set as the dependent variable. The predictor variables included variations in frontal plane GRF and GRF-knee lever arm at time of each peak KAM. In the third model, analyses were repeated with the variations in the KAAI as the dependent variable, and the variations in the average GRF-knee lever arm, average frontal-plane GRF, and stance time as predictor variables.

## Results

Each subject possessed very similar spatiotemporal gait patterns when walking with different footwear concepts (Table 1). The only significant difference was found in the duration of stance phase, where the subjects exhibited a relatively longer stance when wearing the SH shoes compared to the control shoes (*P < 0.001*).

**TABLE 1:** Gait spatiotemporal variables when wearing control shoe compared to the 5° LWI, VS, or SH shoes (F: F-value; P: level of significance; ES: effect size).

Gait variables	Control	5° LWI	VS	SH	F	*P*	ES
Step length (cm)	73.4 ± 8.7	72.8 ± 8.4	73.8 ± 9.4	74.1 ± 9.0	2.024	0.120	0.092
Step width (cm)	10.5 ± 1.9	11.1 ± 2.5	11.2 ± 2.3	10.8 ± 2.1	2.088	0.111	0.095
Stance phase (%)	61.6 ± 1.5	61.8 ± 1.3	61.6 ± 1.5	63.3 ± 1.6	21.383	**0.001**	0.517
Gait velocity (m/s)	1.34 ± 0.22	1.31 ± 0.22	1.33 ± 0.24	1.33 ± 0.24	0.594	0.542	0.029

Bold values indicate statistically significant differences compared with the control shoe condition (P < 0.05).

**FIGURE 2:**
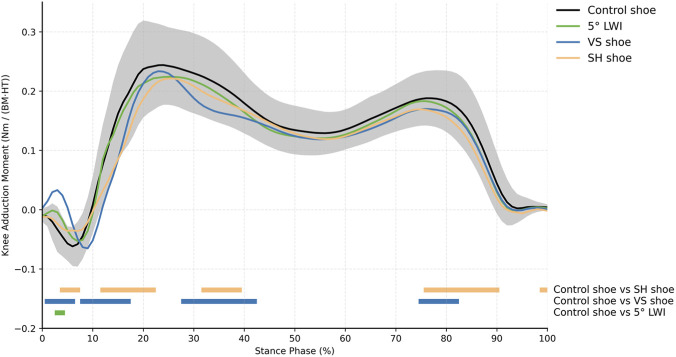
KAM patterns during the stance phase of gait for different shoe concepts. Standard deviation (SD), shown in grey, is only presented for the control shoe. The colour bars denote the outcomes of the SPM1D{F} analysis, indicating significant differences in the KAM patterns between the control shoe and the other footwear throughout the stance phase of gait.

The KAM exhibited a clear double-bump pattern during the stance phase of gait, with the 1st peak KAM generally larger than the 2nd peak KAM (Figure 2). SPM1D{F} indicates significant differences in KAM patterns during the early and late stance phase between control shoes and SH shoes, as well as between control shoes and VS shoes.

**FIGURE 3:**
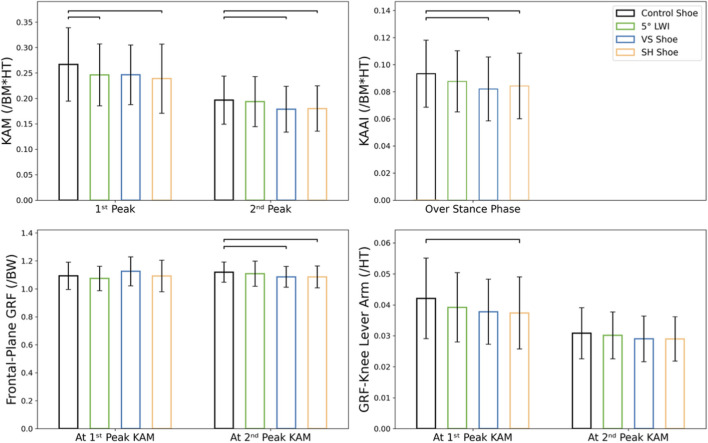
Comparisons of KAM and GRF variables between the 5° LWI, VS, and SH shoes against the control shoe. Bracket label indicates significant pairwise differences.

Statistical analysis revealed significant differences in the 1st peak KAM when walking with different footwear (F = 6.121, *P* = 0.001, ES = 0.234; Figure 3). Compared to control shoes, 5° LWIs and SH shoes reduced the 1st peak KAM by an average of 7.9% (*P* = 0.009) and 10.5% (*P* = 0.001), respectively. In addition, significant differences were observed in the 2nd peak KAM between different footwear (F = 6.015, *P* = 0.001, ES = 0.231). Specifically, VS and SH shoes reduced the 2nd peak KAM by 9.1% (*P* = 0.014) and 8.6% (*P* = 0.032), respectively. Finally, significant differences were observed in KAAI between different footwear (F = 13.187, *P* < 0.001, ES = 0.397), where VS and SH shoes exhibited a reduction of 11.8% (*P* < 0.001) and 9.7% (*P* < 0.001) over the stance phase compared to the control shoes. No significant reduction was observed in the 2nd peak KAM or KAAI while walking with 5° LWIs.

While frontal-plane GRF at the time of the 1st peak KAM did not show significant differences (F = 2.463, *P* = 0.071, ES = 0.110; Figure 3), comparisons between different footwear concepts revealed significant differences at the time of the 2nd peak KAM (F = 5.325, *P* = 0.003, ES = 0.210). Specifically, a reduction of approximately 2.9% was found in the frontal-plane GRF at the time of 2nd peak KAM in both VS (*P* = 0.014) and SH shoes (*P* = 0.014) compared to control shoes. In addition, significant differences were observed in GRF-knee lever arm at the time of the 1st peak KAM (F = 5.732, *P* = 0.002, ES = 0.223), but not at the time of the 2nd peak KAM (F = 2.104, *P* = 0.109, ES = 0.095). Here, the SH shoes reduced the GRF-knee lever arm at the time of 1st peak KAM (*P* < 0.001). The VS shoes also exhibited a clear trend towards reduction, although this was not significant (*P* = 0.056). No significant effect of 5° LWIs was observed in any GRF variables.

At the time of the 1st peak KAM, the knee-COP offset was significantly greater in control shoes compared to VS shoes (*P* < 0.001) and SH shoes (*P* < 0.001) but not compared to 5° LWIs (*P* = 0.087; Table 2). At the time of 2nd peak KAM, all shoes (VS: *P* < 0.001; SH: *P* = 0.008; and 5° LWIs: *P* = 0.014) exhibited reduced knee-COP offset compared to the control shoes.

**TABLE 2:** Kinematic variables when wearing the control shoe, 5° LWIs, VS, and SH shoes.

Kinematic variables	Control	5° LWI	VS	SH	F	*P*	ES
*At 1*st *peak KAM*							
Ankle ever/inversion (°)	−6.3 ± 2.5	−6.2 ± 2.1	−10.1 ± 1.8	−9.4 ± 2.9	35.672	**<0.001**	0.641
Knee-COP offset (cm)	1.3 ± 1.2	0.9 ± 1.2	0.3 ± 1.0	0.7 ± 1.3	18.193	**<0.001**	0.476
Hip-knee-ankle angle (°)	0.9 ± 2.6	1.0 ± 2.5	1.2 ± 2.3	1.0 ± 2.7	0.930	0.432	0.044
Trunk lean angle (°)	0.2 ± 7.1	0.6 ± 8.8	0.0 ± 9.5	1.6 ± 9.0	0.808	0.495	0.039
*At 2*nd *peak KAM*							
Ankle ever/inversion angle (°)	−6.1 ± 2.2	−5.5 ± 2.7	−6.8 ± 3.1	−9.6 ± 2.9	26.523	**<0.001**	0.570
Knee-COP offset (cm)	0.8 ± 1.0	0.4 ± 0.9	0.1 ± 0.8	0.3 ± 0.8	13.022	**<0.001**	0.394
Hip-knee-ankle angle (°)	−0.6 ± 2.4	−0.7 ± 2.3	−0.5 ± 2.2	−0.5 ± 2.4	0.809	0.494	0.039
Trunk lean angle (°)	−0.3 ± 8.3	1.6 ± 7.5	1.0 ± 7.2	0.4 ± 7.1	0.457	0.636	0.022

F: F-value; P: level of significance; ES: effect size.

Bold values indicate statistically significant differences compared with the control shoe condition (P < 0.05).

While no significant differences in hip-knee-ankle angle and trunk lateral lean angle were identified, significant differences were observed in ankle kinematic variables between footwear (Table 2). Subjects showed significantly lager ankle eversion angles at the time of the 1^st^ peak KAM in both VS (*P* < 0.001) and SH shoes (*P* < 0.001) compared to the control shoes. In addition, subjects exhibited significantly larger ankle eversion angles at the time of the 2^nd^ peak KAM in SH shoes (*P* < 0.001) compared to control shoes.

MLR analysis revealed different variables contributing to the variations of KAM variables in VS and SH shoes (Table 3). The GRF-knee lever arm was the dominant contributor to changes in the 1st peak KAM induced by VS shoes. For the SH shoe, however, the frontal-plane GRF also had a strong contributing effect on the 1st peak KAM in addition to the GRF-knee lever arm. GRF-knee lever arm was the main contributor to changes in the 2nd peak KAM in both VS and SH shoes, showing a much larger effect than the frontal-plane GRF. Mean GRF-knee lever arm and stance phase time were the two main contributors to KAAI changes for both VS and SH shoes, where the contributing effects of mean frontal-plane GRF were only minor in both cases.

**TABLE 3:** MLR analysis showing the relationships between variations in KAM parameters, frontal-plane GRF, and GRF-knee lever arm. Z-score standardised coefficients are presented as mean (95% confidence intervals).

Shoe type (compared to control shoe)	Variable	Adjusted *R* ^2^	Coefficients (95% CIs)	t	F	*P*
VS shoe	*1*st *peak KAM*	​	​	​	​	​
	GRF-knee lever arm Frontal-plane GRF	0.956	0.036 (0.032, 0.040) 0.015 (0.012, 0.019)	20.431 8.633	218.7	<0.001
	*2*nd *peak KAM*	​	​	​	​	​
	GRF-knee lever arm Frontal-plane GRF	0.959	0.022 (0.020, 0.024) 0.005 (0.002, 0.007)	21.470 4.536	237.6	<0.001
	*KAAI*	​	​	​	​	​
	Mean GRF-knee lever arm Mean frontal-plane GRF Stance phase time	0.662	0.008 (0.006, 0.011) 0.002 (−0.002, 0.006) 0.005 (0.001, 0.009)	6.430 0.949 2.391	14.04	<0.001
SH shoe	*1*st *peak KAM*	​	​	​	​	​
	GRF-knee lever arm Frontal-plane GRF	0.918	0.020 (0.016, 0.024) 0.022 (0.018, 0.026)	11.045 12.252	112.8	<0.001
	*2*nd *peak KAM*	​	​	​	​	​
	GRF-knee lever arm Frontal-plane GRF	0.955	0.024 (0.021, 0.026) 0.007 (0.004, 0.009)	20.633 6.003	214.4	<0.001
	*KAAI*	​	​	​	​	​
	Mean GRF-knee lever arm Mean frontal-plane GRF Stance phase time	0.842	0.007 (0.005, 0.008) 0.002 (0.000, 0.004) 0.006 (0.004, 0.008)	8.626 1.838 6.696	36.40	<0.001

t: T-value; F: F-value; P: level of significance.

## Discussion

Individuals afflicted with KOA experience persistent symptoms and an elevated risk of comorbidities. Excessive knee loading has been identified as a pivotal mechanical factor in instigating a cascade of arthritic tissue degeneration, as evidenced by its strong association with KOA progression and pain intensity (Lewinson and Stefanyshyn, 2016; Jones et al., 2014). The efficacy of foot orthoses in balancing knee loading during movement and alleviating KOA symptoms remains inconclusive, with conflicting results reported in the literature (Duivenvoorden et al., 2015; Zafar et al., 2020). One potential explanation for this may be found in the insufficient biomechanical effects of such devices throughout the stance phase of gait (Jones et al., 2014). In addressing this knowledge gap, we have developed a novel approach utilising 3D printing technology to engineer shoe soles with specific geometric and stiffness characteristics, towards reducing the KAM. The results of the gait tests indicate that, in comparison with the conventional 5° LWIs which only significantly reduced the 1^st^ peak KAM over neutral control shoes, the 3D-printed shoe concepts might exhibit better biomechanical efficacy, demonstrated by their effects for significantly reducing the magnitude of KAM’s peaks as well as the angular impulse.

5° LWIs can be considered as an appropriate comparator for testing concept efficacy. Higher wedge angles, such as 7° (Hsu et al., 2015) and 10° (Kerrigan et al., 2002), have also been studied previously, but thicker wedges have been clearly associated with ankle discomfort and low usage adherence (Kerrigan et al., 2002). The incorporation of arch supports in LWIs has been proposed as a means of mitigating discomfort for thicker wedges (Xing et al., 2017). However, no significant biomechanical differences were observed between arched and non-arched LWIs (Dessery et al., 2017; Jones et al., 2014). In our study, 5° LWIs reduced the 1st peak KAM by 7.9%. This finding is consistent with several previous studies reporting overall KAM reductions ranging from 5% to 8% (Kakihana et al., 2005; Hinman et al., 2008), up to around 13% in certain study (Hinman et al., 2008). Different from the 5° LWIs, SH shoes exhibited significant mean reductions not only in the 1st peak KAM (10.5%), but also together with VS shoes, significantly reduced the 2nd peak KAM (8.6% and 9.1%). These findings indicate that the 3D-printed shoes, especially the SH shoe, may demonstrate greater immediate reductions in KAM’s peaks from the control shoes, in comparison with the conventional LWIs. In fact, a recent study reported a shoe with a 3D-printed variable stiffness hind-sole component, and preliminarily demonstrated its efficacy in relieving cartilage stress (Chen et al., 2022), which would be consistent with the reduction in KAMs found in our study. Furthermore, while previous studies have predominantly focused on peak KAM reductions during walking, KAAI is an additional surrogate parameter for the force exerted on the medial side of the knee throughout stance phase (Kean et al., 2012) that warrants attention. In this study, it was found that, in contrast to LWIs, both VS and SH shoes significantly reduced the KAAI (11.8% and 9.7%), hence indicating that such shoe sole concepts are effective throughout the loaded phase of gait and not just for reducing peak loading.

The threshold of KAM reduction required to induce meaningful clinical improvement in KOA is not well established. A previous study reported KAM reductions of 5.2% and 6.2% with two types of LWIs but no clear relationship between these biomechanical changes and immediate knee pain relief was observed (Jones et al., 2014). Importantly, however, a 1% increase in peak KAM has been associated with a 6.5-fold increase in the risk of OA progression over long-term follow-up (Miyazaki et al., 2002). In another previous study, a 2% reduction in KAM was defined as the threshold to pre-screen biomechanical responders to LWIs for a randomised clinical trial (Felson et al., 2019). These evidences indicate that even small KAM reductions can play an important role towards enhanced outcomes. However, it remains unknown whether the KAM reductions achieved by the 3D-printed shoes are now able to exceed clinically relevant thresholds for improving KOA symptoms, especially in the consideration of a group of non-KOA subjects were measured in the present study. Future biomechanical studies on these shoe soles with KOA individuals are thus warranted.

LWIs implement a simple but successful wedged shape design to induce ankle eversion (Hinman et al., 2012). Nevertheless, it is important to note that excessive wedging may result in foot pronation. Consequently, conventional LWIs have likely attained their biomechanical efficacy ceiling. Alternatively, previous studies have investigated the orthotic function of VS shoes in modifying dynamic KAMs during walking, which exhibited similar biomechanical effects to LWIs (Erhart-Hledik et al., 2021). It is important to note that VS shoes generally present a flat surface to the foot but induce ankle eversion during the strongly loaded phases of gait, hence serving as both an effective and comfortable alternative to LWIs. VS shoes are therefore associated with a combined effect of decreased medial GRF, lateral shifting of foot COP, as well as valgus thrust of knee induced by ankle eversion (Jenkyn et al., 2011). In our study, in addition to significant ankle eversion, a significant reduction in knee-COP offset distance was observed with our VS shoes, which indicated a lateral shifting of the foot COP with respect to the knee joint centre. Unlike many previous studies that reported a shift of the COP with respect to the ankle or foot (Madden et al., 2017; Jenkyn et al., 2011), the present findings offer a more informative perspective by providing a direct quantification of knee-foot interactions as a kinematic chain. VS shoes have been reported to reduce peak KAMs by approximately 5%–6.6% (Jenkyn et al., 2011; Teoh et al., 2013) in individuals with KOA during walking. Compared to these results, a greater percentage reduction in peak KAM was achieved by our VS shoes in this study. It is undeniable that the difference between KOA and healthy participants could play a role in causing these different biomechanical outcomes. Moreover, these differences plausibly stem from the different lateral-to-medial ratio of sole stiffness between the shoes. While previous studies used a stiffness ratio of around 1.5:1, our VS shoe was designed with a lateral sole stiffness that was twice as high as the medial side. With a greater mediolateral difference in sole stiffness, our VS shoe could induce greater ankle eversion, ultimately leading to a greater reduction in KAM magnitudes. However, greater stiffness ratios are likely to be compromised by increased pain at the ankle. To date, there is currently a lack of evidence to establish the dose effect of this ratio variation in VS shoes on knee loading or ankle/foot pain levels in KOA individuals. Given the potential substitution role of this type of shoe for LWIs, a better understanding of the relationship between its stiffness properties and the resulting biomechanical effects is warranted to inform the optimised design of VS shoe soles.

A significant strength of this study was the utilisation of 3D printing technology to fabricate footwear that incorporates orthotic functionality, characterised by a regionally different sole stiffness design. The gyroid infill’s density in the model can be readily modified to allow alterations in sole stiffness. The 3D printer then fabricates the sole in a single-step moulding process, eliminating the requirement for material splicing. This approach offers clear advantages over traditional manufacturing processes in terms of shoe design revision iteration. In this study, two types of shoes—the VS and SH shoes—were produced. While the biomechanical efficacy of these two shoe designs towards reducing KAM variables were comparable, the underlying mechanisms through which they affected the 1st peak KAM varied significantly, aligning with their distinct sole stiffness characteristics in the hind-region. Specifically, the MLR analysis revealed that the reduction in 1st peak KAM in the VS shoe was more sensitive to shortening of GRF-knee lever arm than to the decrease in frontal-plane GRF. This finding is reasonable and concurs with a previous study (Teoh et al., 2013), given that a reduction in knee-COP offset with the VS shoes would likely shorten the GRF-knee lever arm rather than reduce the magnitude of GRF. On the other hand, the MLR analysis revealed that in the SH shoe, the decrease in frontal-plane GRF contributed slightly more than the lever arm to the reduction of 1^st^ peak KAM. This is likely due to the cushioning effect provided by the overall softness of the hind sole in the SH shoes (Malisoux et al., 2023). In conclusion, the present study has demonstrated the viability of utilising a 3D printing approach for fabricating shoe soles with distinct orthotic functionality, thereby modulating biomechanical characteristics associated with KOA. This approach has the potential to allow fabrication of customised orthotic shoes, tailored to the biomechanical needs of individual patients, through identifying mechanically critical parameters. However, it should be noted that the biomechanical efficacy of current foot orthoses varies among individuals, and the correlation between their clinical and anatomical characteristics, as well as their biomechanical response to shoe design, remains to be elucidated. Moreover, the longevity of such 3D-printed shoe soles needs to be fully investigated. Consequently, future research endeavours may focus on identifying and classifying patient-specific data, with the objective of informing the fabrication process towards individualized 3D-printed orthotic shoes.

It is important to acknowledge three main limitations of this study. Firstly, the study focused on shod gait measurements conducted on healthy subjects, rather than on individuals with KOA. As this was the first study into the development of 3D-printed orthotic technologies, the decision was taken to conduct initial testing on healthy subjects, in order to minimize ethical concerns and facilitate quicker design iteration and modifications. Subsequent studies will include KOA patients to further validate the findings. Secondly, while the KAM serves as a surrogate measure of medial knee loading, it does not directly quantify compressive tibiofemoral loading. The actual changes in tibiofemoral loading that occur with different shoes remain unknown, and investigation into these factors could provide a more comprehensive understanding of the shoe’s biomechanical effects. Finally, ankle discomfort was not assessed while participants were wearing different footwear, as the present study mainly focused on investigating their immediate biomechanical effects. In addition, significant ankle discomfort was primarily reported only in those LWIs with high inclination angles (10°) (Kerrigan et al., 2002). Thus, statistically meaningful differences between the tested footwear in wearing comfort was not expected. However, future studies should quantitatively compare ankle/overall discomfort in individuals with KOA under different shoe conditions, and investigate their potential influnece on wearers’ long-term adherence.

In conclusion, compared to 5° LWIs, the 3D-printed shoe soles demonstrated superior reductions in both peak KAM and KAAI from the control shoes, thereby underscoring their potential as a viable alternative intervention. Future research should explore responses of KOA patients to these shoes and establish optimal design parameters to enhance clinical outcomes.

## Data Availability

The raw data supporting the conclusions of this article will be made available by the authors, without undue reservation.
