# Supplementary material for: 3D-printed shoe soles with structural properties towards reducing knee adduction moments during walking
Source: Front Bioeng Biotechnol. 2026 Jan 20;14:1734486. doi: 10.3389/fbioe.2026.1734486 (PMC12864507; doi:10.3389/fbioe.2026.1734486)
Supplement: Supplementary file 1 [file Table1.docx]

# 3D-Printed Shoe Soles with Structural Properties towards Reducing Knee Adduction Moments Associated with Medial Knee Osteoarthritis

## Supplementary Table:

Table S1: Definitions of gait and biomechanical variables of interest.

|  | Definitions |
| --- | --- |
| *Gait spatiotemporal variables* |  |
| Step length | Distance from heel contact of the contralateral limb to heel contact of the ipsilateral limb along the line of progression. |
| Step width | Distance perpendicular to the line of progression between heel contact of the contralateral limb and heel contact of the ipsilateral limb. |
| Stance phase | Time from heel-strike to toe-off for the ipsilateral limb as a percentage of the gait cycle. |
| Gait velocity | Average velocity throughout the gait cycle. |
| *Kinematic variables* |  |
| Lateral trunk lean angle | Trunk angle in the frontal plane, determined as the line from the mid-point of the pelvis and mid-point of the acromion markers with respect to the vertical. A positive value indicates a lean towards the side of the ipsilateral limb. |
| Hip-knee-ankle angle | Angle formed by the hip, knee, and ankle joint centres in the frontal plane. A positive value indicates knee varus. |
| Knee-COP offset | Mediolateral distance between the vertical projection of the knee joint centre and the foot COP. A positive value indicates lateral offset of the knee. |
| Ankle ev/inversion angle | Ankle angle in the frontal plane. A positive value indicates ankle inversion. |
| *Kinetic variables* |  |
| Frontal-plane GRF | Magnitude of GRF in frontal plane. |
| GRF-knee lever arm | Perpendicular distance between the GRF and knee joint centre in the frontal plane. |
